# Integration of tumor microenvironment and metabolic signatures reveals TMPI-defined prognostic and immunotherapy-relevant phenotypes in breast cancer

**DOI:** 10.3389/fphar.2026.1852654

**Published:** 2026-06-22

**Authors:** Zhong Huang, Shuhui Lin, Ying Liang, Muhammad Khan, Weirui Chen, Xuefeng Li, Xiaolan Liu, Yunyu Wu, Jiacai Ye

**Affiliations:** 1 Department of Radiation Oncology, The Second Affiliated Hospital of Guangzhou Medical University, Guangzhou, Guangdong, China; 2 Department of Radiation Oncology, Guangzhou Institute of Cancer Research, The Affiliated Cancer Hospital, Guangzhou Medical University, Guangzhou, Guangdong, China; 3 Department of Radiation Oncology, Shenzhen Nanshan People’s Hospital, Shenzhen, Guangdong, China; 4 Guangzhou Liwan Maternal & Child Health Hospital, Guangzhou, Guangdong, China; 5 Department of Gynecologic Oncology, Guangzhou Institute of Cancer Research, The Affiliated Cancer Hospital, Guangzhou Medical University, Guangzhou, Guangdong, China

**Keywords:** cancer metabolism, cancer-associated fibroblasts, immunotherapy, transcriptomic analysis, tumor microenvironment

## Abstract

**Background:**

Breast cancer heterogeneity presents a significant challenge for effective diagnosis and treatment. A deeper understanding of the tumor microenvironment (TME) and its metabolic dynamics is essential for addressing this complexity. This study explored the interplay between TME components and metabolic profiles in breast cancer using RNA sequencing data from both bulk and single-cell analyses.

**Methods:**

Transcriptomic data and corresponding clinical information for breast cancer patients were obtained from TCGA and GEO databases. qPCR and immunohistochemistry (IHC) were performed for experimental validation. Data analyses were conducted using R software.

**Results:**

A high-risk phenotype was identified based on 36 interconnected genes primarily associated with the TME and metabolic processes. This phenotype demonstrated enrichment of nicotinamide adenine dinucleotide (NAD^+^) metabolism and was associated with reduced T- and NK-cell activity within the TME, contributing to poor prognosis in HER2 and Luminal B breast cancer subtypes. Using Least Absolute Shrinkage and Selection Operator (LASSO) regression, a 23-gene risk model (TMPI) was established, which further refined prognostic stratification and highlighted elevated risk in HER2 and Luminal B contexts. Immune and stromal analyses, along with single-cell validation, revealed that key NAD metabolism genes (NMNAT2 and QPRT) and the adenosine pathway enzyme NT5E were predominantly expressed in stromal and malignant cell populations, including endothelial cells and fibroblasts. qPCR validation confirmed subtype-specific expression patterns, with NMNAT2 and QPRT upregulated in ER-positive cells and NT5E enriched in triple-negative breast cancer (TNBC) cells. IHC analysis further validated their increased expression in tumor tissues compared to adjacent normal tissues. Importantly, TMPI was consistently associated with immune-desert and immune-excluded tumor microenvironment states across immunotherapy cohorts and demonstrated therapy-dependent associations with anti–PD-1 and anti–PD-L1 treatment response patterns, suggesting that NAD metabolism-associated immune states may influence immunotherapy sensitivity in a context-dependent manner.

**Conclusion:**

These findings provide insights into the interplay between metabolism and the tumor microenvironment in breast cancer. TMPI captures the prognostically relevant dimension of TME–metabolism crosstalk and may serve as a useful framework for patient stratification, characterization of tumor immune phenotypes, and therapeutic targeting, particularly in HER2+ and Luminal B breast cancers.

## Introduction

Breast cancer remains the most commonly diagnosed malignancy among women worldwide, with an estimated 2.3 million new cases reported annually (Sung et al., 2021). Despite advances in molecular classification and targeted therapies, breast cancer continues to exhibit substantial intertumoral and intratumoral heterogeneity, leading to variable clinical outcomes and therapeutic responses (Yersal and Barutca, 2014; Jallah et al., 2023). While established subtypes such as hormone receptor-positive and HER2-positive breast cancers have benefited from tailored treatments, significant variability persists within these groups, underscoring the need for more precise prognostic and therapeutic biomarkers (Parker et al., 2023; Fallahpour et al., 2017).

In recent years, the tumor microenvironment (TME) has emerged as a critical determinant of cancer progression and therapeutic response (Domínguez-Cejudo et al., 2023). Composed of diverse immune and stromal cell populations, the TME is partly shaped by tumor-intrinsic features, including molecular subtype-specific signaling programs and metabolic activity (Anderson and Simon, 2020). Accordingly, distinct breast cancer subtypes exhibit characteristic microenvironmental profiles, reflecting differences in immune infiltration and stromal composition (Li et al., 2022). However, this relationship is not unidirectional. Increasing evidence indicates that the TME actively contributes to tumor heterogeneity by imposing selective pressures that influence tumor cell behavior, clonal evolution, and therapeutic resistance.

In this context, the TME should be considered an active regulator of tumor biology rather than merely a passive consequence of tumor subtype. Variability in immune cell infiltration has been associated with differential survival outcomes in breast cancer, underscoring the functional impact of TME composition on disease progression (Sun et al., 2023). Moreover, stromal and immune components can reshape tumor phenotypes through signaling interactions and metabolic constraints, thereby reinforcing tumor diversity. These findings highlight a dynamic and reciprocal relationship in which tumor cells and the TME co-evolve, jointly driving disease progression and influencing therapeutic outcomes (Kim et al., 2022; Mittal et al., 2018).

A key mechanism underlying this bidirectional interaction is cellular metabolism, which serves as a functional interface linking tumor cells with their microenvironment (Kim et al., 2022; Mittal et al., 2018). Metabolic reprogramming in cancer not only supports tumor growth and survival but also modulates the activity and differentiation of immune and stromal cells, contributing to the establishment of an immunosuppressive microenvironment (Ogrodzinski et al., 2017; Feng et al., 2019; Locasale, 2012). In particular, metabolic pathways involved in nucleotide biosynthesis and energy homeostasis have been implicated in regulating immune cell function and therapeutic responsiveness (Wei et al., 2020; Rabold et al., 2017; Lopes-Coelho et al., 2018). Despite these advances, the extent to which metabolic processes coordinate with TME components to shape breast cancer heterogeneity and clinical outcomes remains insufficiently defined.

In this study, we hypothesized that coordinated crosstalk between tumor metabolism and TME components defines clinically relevant molecular subtypes of breast cancer. To address this, we integrated bulk and single-cell transcriptomic analyses to systematically identify genes involved in both metabolic and TME-related processes and to characterize their interactions at the network level. We further developed a prognostic index based on these crosstalk genes and evaluated its association with immune landscape, clinical outcomes, and immunotherapy response. Collectively, this study provides a comprehensive framework linking metabolic reprogramming with TME dynamics in breast cancer and identifies potential biomarkers and therapeutic targets for improving patient stratification and treatment strategies.

## Methods & materials

### Definition of TME-Metabolism gene space

To systematically investigate the interaction between tumor metabolism and the tumor microenvironment (TME), we first constructed a comprehensive gene space encompassing both biological domains. A total of 4,061 TME-related genes were curated from established computational frameworks, including xCell, CIBERSORT, and MCP-counter, along with previously reported immune-related gene signatures and single-cell datasets (Aran et al., 2017; Newman et al., 2015; Becht et al., 2016; Chifman et al., 2016; Rooney et al., 2015; Tirosh et al., 2016). In parallel, 944 metabolism-associated genes were obtained from KEGG-based metabolic pathways in the Molecular Signatures Database (Supplementary Material Table S1). After removing redundancies (n = 211 genes), a unified set of TME-Metabolism related genes (n = 3,850 genes) was established to define the analytical gene space for subsequent analyses.

### Data acquisition and harmonization

Gene expression profiles and corresponding clinical data for breast cancer were obtained from The Cancer Genome Atlas (TCGA-BRCA) using the TCGAbiolinks R package, including 1,069 tumor samples and 113 normal breast tissues. RNA-seq data generated using the STAR–Counts workflow were retrieved from the Genomic Data Commons (GDC). FPKM-normalized expression values were extracted from the *fpkm_unstranded* assay and log2-transformed [log2(FPKM +1)] for downstream analyses. Ensembl gene identifiers were mapped to official gene symbols using the GENCODE human annotation. Redundant gene symbols were collapsed by retaining a single representative per gene based on the highest average expression across samples, resulting in a non-redundant gene-level expression matrix.

For external validation, the GEO cohort (GSE20685, n = 327; platform GPL570) was included. To ensure comparability across platforms, RNA-seq and microarray datasets were harmonized through gene intersection and batch-effect correction using the ComBat algorithm. Expression values were log-transformed, and cohort-specific matrices were reconstructed following correction. This integration enabled robust cross-platform validation of downstream analyses.

### Identification of TME-Metabolism crosstalk genes

Differential expression analysis was performed between tumor and normal tissues to identify TME- and metabolism-related genes with significant alterations (|logFC| ≥ 1, FDR <0.05). Prognostic relevance was assessed using univariate Cox regression analysis. To identify functional interactions between these gene sets, protein–protein interaction (PPI) analysis was conducted using STRING, version 11.5 (https://string-db.org/). This approach enabled the extraction of a core network of genes representing potential crosstalk between metabolic processes and TME components, forming the basis for subsequent clustering and modeling analyses.

### Molecular subtyping via NMF

To capture biological heterogeneity driven by TME–metabolism interactions, Non-negative Matrix Factorization (NMF) clustering was applied to the expression profiles of the identified crosstalk genes using the *NMF* R package (Gaujoux and Seoighe, 2010). The algorithm was implemented with 100 iterations under the “brunet” method to ensure clustering stability. The optimal number of clusters (k = 2–10) was determined by evaluating cophenetic correlation coefficients, dispersion, and silhouette width metrics. The final clustering solution was selected based on maximal stability and interpretability of the consensus matrix. This unsupervised approach enabled the identification of molecular subtypes characterized by distinct immune and metabolic features.

### Construction of the TME–Metabolism Prognostic Index (TMPI)

To refine the crosstalk gene network into a clinically applicable prognostic model, Least Absolute Shrinkage and Selection Operator (LASSO) Cox proportional hazards regression was performed using the *glmnet* R package (Ishwaran et al., 2014). The expression matrix of prognostically significant crosstalk genes was used as input, with overall survival time and status as response variables. Model optimization was achieved through ten-fold cross-validation to determine the optimal penalty parameter (λ), selecting the value that minimized the partial likelihood deviance. Genes with non-zero coefficients at the optimal λ were retained to construct the prognostic signature.

A risk score (termed TMPI) was calculated for each patient as a linear combination of gene expression levels weighted by their corresponding LASSO-derived coefficients. Patients were stratified into high- and low-risk groups based on the median TMPI score. The prognostic performance of the model was evaluated using Kaplan–Meier survival analysis with log-rank tests, and time-dependent receiver operating characteristic (ROC) curves were generated using the *survivalROC* R package to assess predictive accuracy. The robustness of the model was further validated in an independent GEO cohort.

### Single-cell transcriptomic analysis

To validate the cellular origins and functional relevance of TME–metabolism crosstalk genes, single-cell RNA sequencing datasets were analyzed using the Tumor Immune Single-cell Hub (TISCH) platform (Sun et al., 2021), which provides standardized preprocessing and cell-type annotation based on the MAESTRO pipeline (Wang et al., 2020). We examined multiple breast cancer datasets, including primary (BRCA_GSE148673 and BRCA_EMTAB8107) and metastatic samples (BRCA_GSE150660), along with additional large-scale datasets (GSE161529 and GSE176078). Expression patterns of the TME–metabolism gene signature and key genes (NMNAT2, QPRT, and NT5E) were evaluated across major cell populations, including malignant cells, fibroblasts, and immune cells, to define their cellular distribution within the tumor microenvironment.

### Tumor microenvironment characterization

The relative abundance of 22 immune cell types was estimated using the CIBERSORT algorithm (Newman et al.). Tumor purity, along with immune and stromal scores, was assessed using the *ESTIMATE* R package (Yoshihara et al., 2013). The MCP-counter algorithm was applied to quantify the absolute abundance of major immune and stromal cell populations within the TME (Becht et al., 2016). Additionally, TIMER v2.0 was used to evaluate the association between immune cell infiltration and the expression of key genes (NMNAT2, QPRT, and NT5E) in the TCGA-BRCA cohort (Li et al., 2020). All analyses were based on bulk tumor gene expression data.

### Functional enrichment analysis

The “gsva” package of R was used to estimate the enrichment of the hallmark cancer and KEGG pathways operating between the clusters. ShinyGO 0.80 (http://bioinformatics.sdstate.edu/go/) was used to assess the enrichment of 23 TMPI genes in Gene Ontology (GO) and KEGG pathways (Ge et al., 2020).

### Immunotherapy response assessment

TMPI response associations were first assessed in the breast cancer anti–PD-1-treated dataset GSE194040 (Pembrolizumab; n = 69). Associations between TMPI and immune phenotypes (inflamed, excluded, and desert) were further evaluated in an independent breast cancer TIME dataset, GSE177043 (anti–PD-1-treated; n = 43). External validation was subsequently performed using the IMvigor210 cohort (urothelial carcinoma; n = 348; immune checkpoint inhibitor = anti–PD-L1). The impact of individual NAD metabolism-related genes on immunotherapy response was additionally investigated using the ROC Plotter immunotherapy datasets (https://rocplot.org/immune), encompassing 1,434 cancer patients treated with anti–PD-1, anti–PD-L1, or anti–CTLA-4 therapies (Kovács et al., 2023).

### Cell lines and culture conditions

Human breast cancer cell lines, including MCF-7, MDA-MB-231, T47D, and BT549, and the non-cancerous breast epithelial cell line MCF-10A were procured from the Type Culture Collection of the Chinese Academy of Sciences (Shanghai, China). The cells were grown in Dulbecco’s modified Eagle’s medium (DMEM) supplemented with 10% fetal bovine serum (FBS), 100 U/mL penicillin, and 100 μg/mL streptomycin. Cultures were maintained at 37 °C in a humidified atmosphere containing 5% CO2. The cell lines were routinely authenticated by monitoring cellular morphology and screening for *mycoplasma* contamination to ensure the reliability of the experimental results.

### Quantitative real-time PCR (qPCR)

Total RNA was isolated using TRIzol reagent (Takara, Otsu, Japan) following the manufacturer’s protocol. The extracted RNA was reverse-transcribed into complementary DNA (cDNA) which served as a template for subsequent qPCR analysis. Amplification was performed using SYBR Green PCR Master Mix (Takara, Otsu, Japan). GAPDH was used as an internal reference gene to normalize the mRNA expression levels. Comparative expression levels were analyzed by calculating fold changes relative to untreated controls. Detailed information regarding primer sequences is provided in Supplementary Material Table S2.

### Clinical specimens

Tumor and adjacent normal tissue samples were collected from 10 patients with breast cancer between December 2023 and August 2024 at the Department of Radiation Oncology, Guangzhou Institute of Cancer Research, Affiliated Cancer Hospital, Guangzhou Medical University. Written informed consent was obtained from all participants, and the study was approved by the Internal Review and Ethics Boards of the Affiliated Cancer Hospital and Institute of Guangzhou Medical University.

### Immunohistochemical analysis

Immunohistochemistry (IHC) was performed on 4 µm sections of formalin-fixed, paraffin-embedded tumors and adjacent normal tissues obtained from patients with breast cancer. The sections were deparaffinized using xylene and graded ethanol, followed by antigen retrieval in citrate buffer (pH 6.0) using microwave heating. To block endogenous peroxidase activity, the sections were treated with 0.3% hydrogen peroxide and subsequently incubated with 5% bovine serum albumin (BSA) in phosphate-buffered saline (PBS) to minimize nonspecific binding. Primary antibodies, including anti-NMNAT2 (Affinity, #DF13581, rabbit, 1:200), anti-NT5E (Proteintech, #12231-1-AP, rabbit, 1:500), and anti-QPRT (Proteintech, #25174-1-AP, rabbit, 1:200), were applied and sections were incubated overnight at 4 °C. The following day, the sections were incubated with biotinylated secondary antibodies at room temperature, and staining was performed using 3,5-diaminobenzidine (DAB) as the chromogen. Hematoxylin was used as the counterstain. Staining intensity was graded on a scale of 0–3 (0: no staining, 1: weak, 2: moderate, 3: strong), while the proportion of positively stained cells was scored from 0 to 4 (0, <5%, 1:5%–25%, 2:26%–50%, 3:51%–75%; 4, >75%). The final IHC score was calculated by multiplying the intensity and frequency scores. For tissues exhibiting heterogeneity, individual regions were scored separately and the results were combined to produce the final score.

### Statistical analysis

R v4.0.3 (http://www.r-project.org) was used for all statistical analyses. Categorical variables were compared using the chi-square test, while two or more groups were compared using the Student’s t-test and ANOVA/Kruskal–Wallis tests. Survival analysis was performed using the Kaplan-Meier method with log-rank test. Univariate and multivariate factor analyses were performed using the Cox regression hazard models. Spearman/Pearson correlation tests were used to assess correlations.

## Results

### Identification of TME–Metabolism crosstalk genes associated with prognosis

**FIGURE 1:**
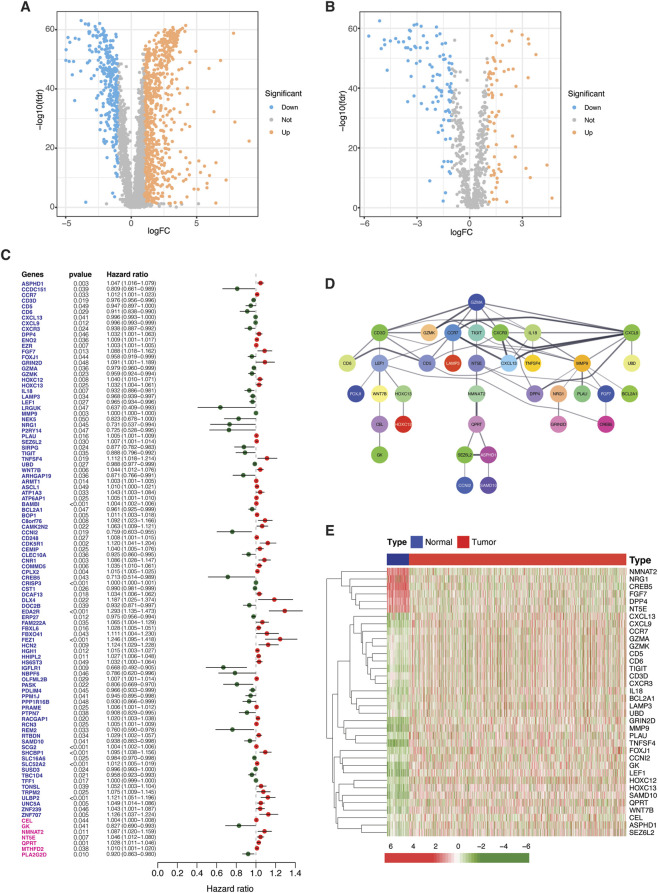
Identification of TME and metabolism crosstalk. **(A)** Volcano plots shows differential expression analysis of TME- and **(B)** metabolism-related genes (TME-Metab DEGs). LogFC = 1, FDR <0.05. **(C)** Forest plot depicts Uni-Cox regression analysis. Blue and Pink represent TME and Metab DEGs, respectively. **(D)** Protein-protein interaction (PPI) network illustrates protein-level interactions of TME-Metab DEGs (interaction score = 0.4). **(E)** Heatmap shows the expression pattern of TME-Metab DEGs between TCGA BRCA normal (n = 113) and tumor samples (n = 1,069).

To delineate the interplay between tumor metabolism and the tumor microenvironment (TME), we analyzed the expression of metabolism-related (n = 944) and TME-associated genes (n = 3,950) across TCGA-BRCA tumor (n = 1,069) and normal tissues (n = 113). Differential expression analysis (|logFC| ≥ 1, FDR <0.05) identified 1035 TME-related and 167 metabolism-related genes with significant alterations between tumor and normal samples (Figures 1A,B).

To determine their clinical relevance, univariate Cox regression analysis (P < 0.05) identified 95 TME-related and seven metabolism-related genes significantly associated with patient prognosis (Figure 1C). Integration of these prognostically relevant genes using protein-protein interaction analysis (STRING, interaction score >0.4) revealed a tightly connected crosstalk network comprising 36 genes, representing coordinated interactions between metabolic pathways and microenvironmental components (Figure 1D). Expression profiling of these genes demonstrated a predominant upregulation pattern in tumor tissues, with 30 genes upregulated and six downregulated (Figure 1E), suggesting a concerted activation of TME–metabolism programs in breast cancer. Collectively, these findings identify a core set of interacting genes that link metabolic reprogramming with microenvironmental dynamics and are strongly associated with clinical outcomes in breast cancer.

### Impact of TME-Metabolism crosstalk on tumor microenvironment remodeling

**FIGURE 2:**
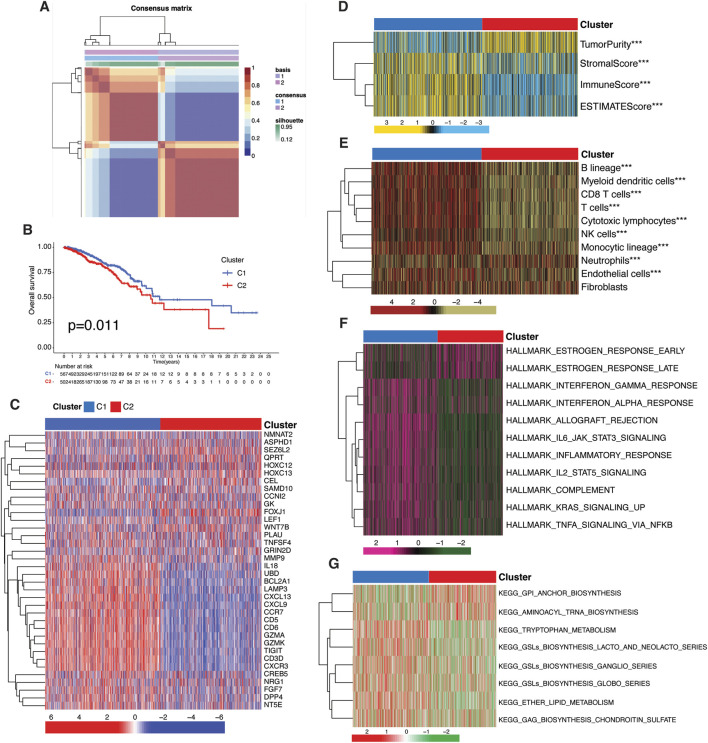
Molecular subtypes based on TME-Metab cross-talk. **(A)** Consensus clustering matrix. **(B)** Kaplan-Meier curves comparing overall survival between clusters. **(C)** Heatmap displaying the expression of 36 TME-Metab DEGs. **(D)** Heatmap depicting the enrichment results of the ESTIMATE algorithm. **(E)** Single-cell gene set enrichment analysis (ssGSEA) of immune cell infiltration between clusters using the MCP-counter algorithm. Significance levels represented as: *P < 0.05; **P < 0.01; ***P < 0.001. **(F)** Heatmaps presenting GSVA enrichment scores of hallmark cancer pathways, and **(G)** KEEG metabolic pathways within the clusters.

To investigate the biological implications of TME–metabolism crosstalk, patients were stratified based on the expression of the 36 crosstalk genes using Non-negative Matrix Factorization (NMF). Evaluation of clustering stability identified k = 2 as the optimal solution, defining two distinct molecular subtypes (Figure 2A). These subtypes exhibited significantly different overall survival outcomes (p = 0.011), indicating the prognostic relevance of TME–metabolism interactions (Figure 2B).

Distinct expression patterns of crosstalk genes were observed between the two clusters (Figure 2C). Cluster one was characterized by elevated expression of immune-related genes, including T- and NK-cell markers (CD3D, CD3E, CD6, CXCR3, CCR7, TIGIT, GZMA, and GZMK), whereas Cluster two showed enrichment of metabolism-associated genes such as NMNAT2, QPRT, NT5E, CEL, and GK. Consistent with these expression patterns, tumor microenvironment analysis revealed that Cluster two exhibited higher tumor purity and reduced immune and stromal infiltration, while Cluster one demonstrated an immune-enriched phenotype (Figure 2D). Further characterization using MCP-counter confirmed a marked depletion of immune and endothelial cell populations in Cluster 2, with neutrophils being the only relatively enriched immune component (Figure 2E).

Pathway analysis further highlighted functional differences between the clusters. Cluster two showed suppression of immune-related pathways alongside activation of estrogen receptor signaling (Figure 2F), whereas metabolic pathways, including glycosylphosphatidylinositol (GPI) anchor biosynthesis and aminoacyl-tRNA biosynthesis, were enriched. In contrast, Cluster one demonstrated enrichment of glycosphingolipid biosynthesis pathways (Figure 2G).

Collectively, these findings define two biologically distinct subtypes driven by TME-metabolism crosstalk: an immune-enriched subtype and a metabolically active, immune-depleted subtype, highlighting the role of coordinated metabolic and microenvironmental interactions in shaping tumor behavior and clinical outcomes in breast cancer.

### Clinical relevance of TME-Metabolism subtypes across breast cancer classes

**FIGURE 3:**
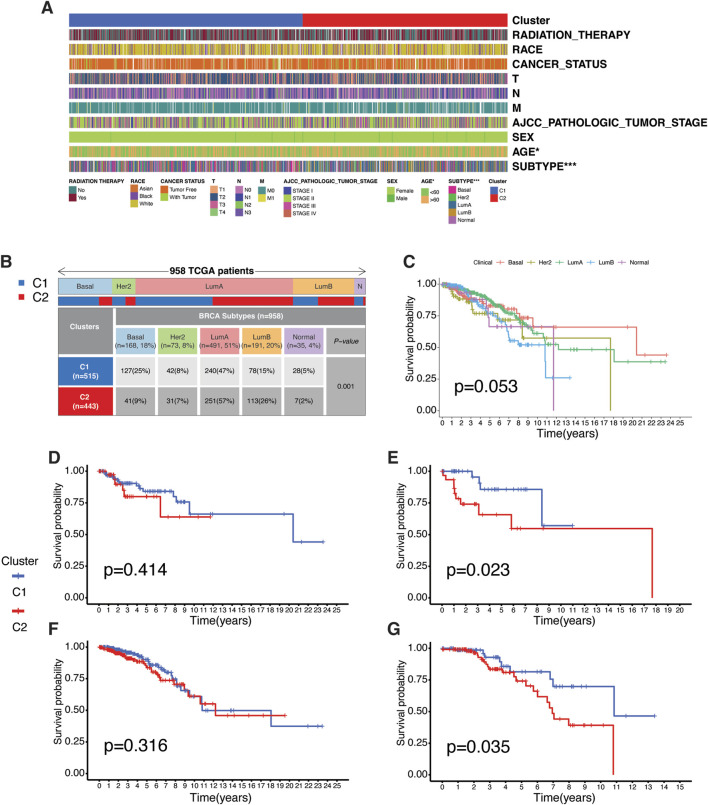
Clinical implications of TME-Metab cross-talk. **(A)** Heat map illustrating the distribution of clinical features between clusters. *P < 0.05; ***P < 0.001. **(B)** Heatmap and table showing the distribution of the BRCA PAM50 subtypes between clusters. **(C)** Kaplan-Meier curves comparing overall survival among BRCA molecular subtypes. **(D)** Kaplan-Meier curves comparing overall survival between clusters in TNBC. **(E)** HER2, **(F)** Luminal A and **(G)** luminal B subtypes. Statistical significance was set at P < 0.05.

To assess the clinical significance of the identified TME-metabolism subtypes, we compared clinicopathological features between the two clusters. No significant differences were observed in race, tumor stage, or therapeutic response (Figure 3A). However, notable differences were identified in age distribution and intrinsic molecular subtypes. Patients in Cluster two were generally older, with a higher proportion of individuals aged over 60 years. In terms of molecular subtype composition, Cluster one was enriched in basal-like and normal breast cancer subtypes, whereas Cluster two showed a higher proportion of luminal subtypes, particularly Luminal B (Figure 3B).

Although basal-like breast cancer is typically associated with aggressive behavior and poor prognosis, Cluster 1—which contained a higher proportion of basal-like cases—demonstrated improved survival outcomes. This observation suggests that tumor immune microenvironment (TIME) and metabolic characteristics captured by the TME–metabolism signature may influence prognosis beyond intrinsic molecular subtype classification alone. Further stratified survival analyses demonstrated that the TME–metabolism subtypes showed the strongest prognostic stratification in HER2+ and Luminal B breast cancers, whereas no significant survival differences between clusters were observed in basal-like or Luminal A tumors (Figures 3C–G). These findings suggest subtype-specific differences in the prognostic relevance of TME–metabolism crosstalk. Consistent with our observations, recent studies have reported that metabolically active breast cancer states with poorer prognosis are enriched in Luminal B and HER2 subtypes and are associated with proliferative signaling together with reduced immune cell infiltration, including impaired T/NK-cell-associated immune activation, whereas immune-activated states are more frequently observed in Luminal A tumors (Cheng et al., 2025).

### LASSO-based refinement of crosstalk genes and construction of TMPI

**FIGURE 4:**
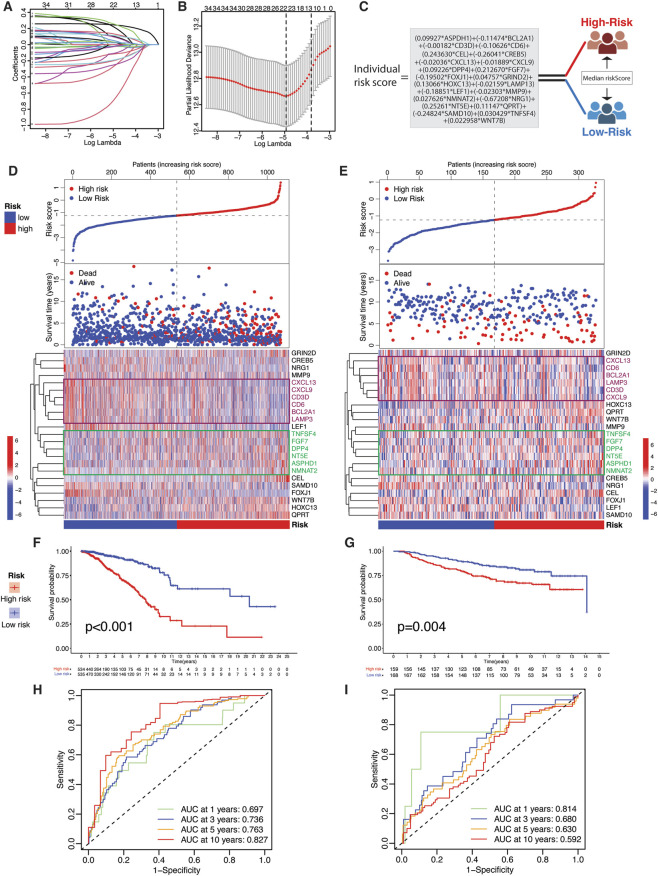
Construction and validation of TME-Metab prognostic index. **(A)** Utilization of LASSO regression on the 36 TME-Metab genes. **(B)** Implementation of cross-validation to fine-tune parameter selection within the LASSO regression framework. **(C)** Visualization illustrating the delineation of BRCA risk subgroups based on the median score derived from the risk formula. **(D)** Presentation of Risk-Score plot, survival plot, and heatmap depicting the expression of risk genes among risk subgroups within both the training dataset (TCGA BRCA) and **(E)** validation dataset (Geo ID: GSE20685). **(F)** Examination of Kaplan-Meier curves to assess the difference in overall survival between risk subgroups within both the TCGA and **(G)** GEO cohorts. **(H)** Assessment of time-dependent receiver operating characteristic (ROC) curves and area under curve (AUC) analyses to evaluate the predictive efficiency of the risk score within both the TCGA and **(I)** GEO cohorts.

To improve the clinical applicability of the 36 TME–metabolism crosstalk genes, LASSO Cox regression analysis was performed in the TCGA-BRCA cohort as the training set, with independent validation in the GEO cohort GSE20685. This approach reduced the 36-gene network to a 23-gene prognostic signature, termed the TME–Metabolism Prognostic Index (TMPI) (Figures 4A,B).

The TMPI risk score for each patient was calculated using the expression levels of the 23 genes weighted by their corresponding regression coefficients (Figure 4C). Based on the median risk score, patients were stratified into high- and low-risk groups in both the training and validation cohorts. Risk distribution plots showed that death events were more frequent in the high-risk group in both TCGA and GSE20685, supporting the prognostic relevance of the model across cohorts (Figures 4D,E). The expression heatmaps further revealed two major functional patterns within the 23-gene signature. One group, largely composed of T-cell-related genes (highlighted in red), was downregulated in the high-risk subgroup, whereas a second group, enriched for metabolism-related and associated genes, was upregulated (highlighted in green) (Figures 4D,E). This expression pattern suggests that the high-risk subgroup is characterized by coordinated metabolic activation together with suppression of T-cell-associated programs, a distinction that informed subsequent functional analyses.

Kaplan–Meier survival analysis demonstrated significantly worse overall survival in the high-risk subgroup in both the TCGA training cohort and the GEO validation cohort (Figures 4F,G). Time-dependent ROC analysis further supported the predictive performance of TMPI, with AUC values ranging from 0.697 to 0.827 in TCGA and from 0.814 to 0.592 in the validation dataset across different time points (Figures 4H,I). Variability in temporal AUC patterns between cohorts may reflect differences in sample size, follow-up duration, event distribution, and clinical composition. Notably, the TCGA cohort contained a substantially larger number of patients (n = 1,069), was predominantly composed of early-stage (Stage I–II) breast cancers, and included extended long-term follow-up of approximately 25 years. In contrast, the GEO validation cohort was smaller (n = 375), clinically distinct, and had a relatively shorter follow-up duration of approximately 15 years, which may influence the stability of late time-point ROC estimation. Overall, these findings support the prognostic value of TMPI across independent breast cancer cohorts.

### TMPI-based stratification refines clinical and TME heterogeneity beyond NMF clusters

**FIGURE 5:**
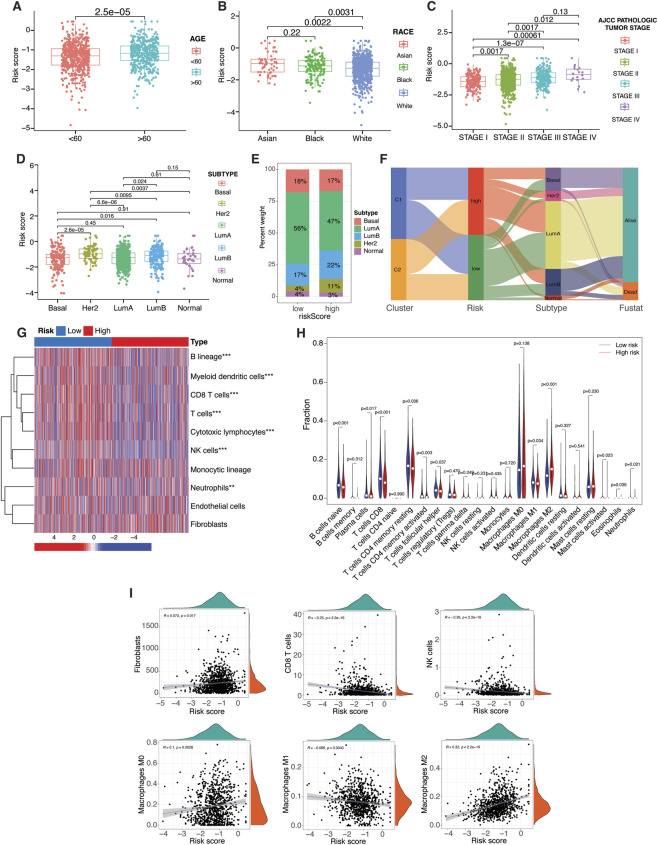
Clinical and immunological features of TME-Metab prognostic index. **(A)** Bar plot showing the difference in risk score between age, **(B)** race, **(C)** clinical stage groups, and **(D)** molecular subtypes. **(E)** Percent of each molecular subtype in the risk subgroups. **(F)** Sankey diagram presenting the correlation between clusters, risk subgroups, BRCA molecular subtypes and survival outcome. **(G)** Single-cell gene set enrichment analysis (ssGSEA) of immune cell infiltration between clusters using the MCP-counter algorithm. Significance levels represented as: *P < 0.05; **P < 0.01; ***P < 0.001. **(H)** Violin plot of abundance of 22 subtypes of immune cells in risk subgroups based on CIBERSPRT algorithm. **(I)** Spearman’s correlation between infiltration of immune cells and TME-Metab riskScore in TCGA SBRCA cohort. P value <0.05 was considered significant.

To further evaluate the clinical relevance of TME-metabolism interactions, we examined how TMPI-based stratification refines the characteristics observed in the original clusters. While age-related trends were consistent with cluster-level observations (Figure 5A), additional distinctions emerged at the TMPI subtype level. Notably, higher TMPI scores were observed in Asian patients compared to Caucasian patients (Figure 5B), and a progressive increase in TMPI scores was evident across tumor stages I to IV (Figure 5C). Subtype-specific analysis revealed that the HER2 subtype exhibited the highest TMPI scores, followed by Luminal B, consistent with the earlier observation that TME–metabolism crosstalk exerts the strongest prognostic impact in these subtypes (Figures 5D,E). However, Sankey diagram analysis demonstrated that TMPI subtypes were not directly aligned with the original clusters, showing a relatively even distribution of cluster membership within each TMPI subtype (Figure 5F). This highlights that TMPI captures the prognostically relevant aspect of TME-metabolism crosstalk.

At the microenvironmental level, refinement of the gene set from 36 to 23 genes altered the observed TME composition. The endothelial cell enrichment seen in cluster-based analysis was diminished in the TMPI subtypes, whereas fibroblast enrichment became more prominent in the high-risk TMPI subtype (Figure 5G). Monocytic lineage cells and neutrophils also showed variable distribution across TMPI subtypes. Importantly, suppression of T and NK cell populations remained a consistent feature of the high-risk TMPI subtype, as confirmed by CIBERSORT analysis (Figure 5H). In addition, macrophage polarization patterns indicated reduced M1 and increased M2 phenotypes, further supporting an immunosuppressive microenvironment. Correlation analysis reinforced these findings, demonstrating significant associations between TMPI scores and immune/stromal cell populations (Figure 5I). These results indicate that TMPI subtypes refine clinical and microenvironmental features, defining a high-risk phenotype marked by T- and NK-cell suppression within an immunosuppressive TME, along with M2 macrophage polarization and fibroblast enrichment.

### Single-cell analysis reveals cellular basis of TMPI-Defined phenotypes

**FIGURE 6:**
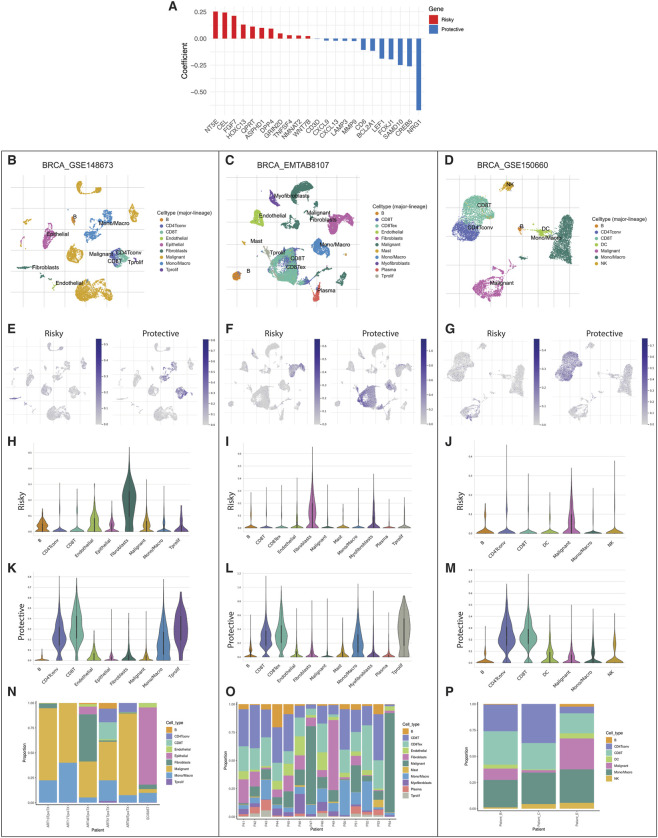
Validation of TME-Metab prognostic index at single cell level. **(A)** Bar plot depicting the regression coefficients of the 23 TME-Metab prognostic index (TMPI) genes. **(B–D)** Umap plots showing main cell types in breast cancer single-cell datasets. **(E–G)** Umap plots displaying the mean expression of Risky (n = 11) and Protective (n = 12) genes of TMPI. **(H–J)** Bubble plots depicts the mean expression of Risky and **(K–M)** Protective genes in each cell type. **(N–P)** Proportion of each cell type in each single-cell dataset.

To further investigate the cellular context of TMPI-associated genes, the 23-gene signature was analyzed at single-cell resolution. Based on their contribution to prognosis, genes were classified into risky and protective groups (Figure 6A). The risky group (n = 11) was predominantly enriched for metabolism-related genes, whereas the protective group (n = 12) was associated with T-cell infiltration and function. Three breast cancer single-cell datasets from the TISCH database were analyzed, including primary tumors (BRCA_GSE148673: n = 6; 10,359 cells and BRCA_EMTAB8107: n = 14; 33,043 cells) and metastatic samples (BRCA_GSE150660: n = 3; 10,605 cells) (Figures 6B–D). These datasets were uniformly processed using the MAESTRO pipeline and annotated by the TISCH platform (Feng et al., 2019; Locasale, 2012).

Expression analysis revealed that risky genes were primarily enriched in fibroblasts and malignant cells, whereas protective genes were predominantly expressed in T-cell populations and monocyte/macrophage lineages (Figures 6E–M). Notably, samples with increased infiltration of fibroblasts and malignant cells—such as ART46TpreTX (BRCA_GSE148673), P49 and P54 (BRCA_EMTAB8107), and Patient_E (BRCA_GSE150660)—exhibited markedly reduced or negligible CD8^+^ T-cell presence (Figures 6N–P). These findings indicate that TMPI-associated risk genes are linked to stromal and tumor cell populations and are associated with reduced T-cell infiltration, consistent with an immunosuppressive tumor microenvironment.

### Functional annotation highlights NAD metabolism as a key axis of TMPI-Defined phenotypes

**FIGURE 7:**
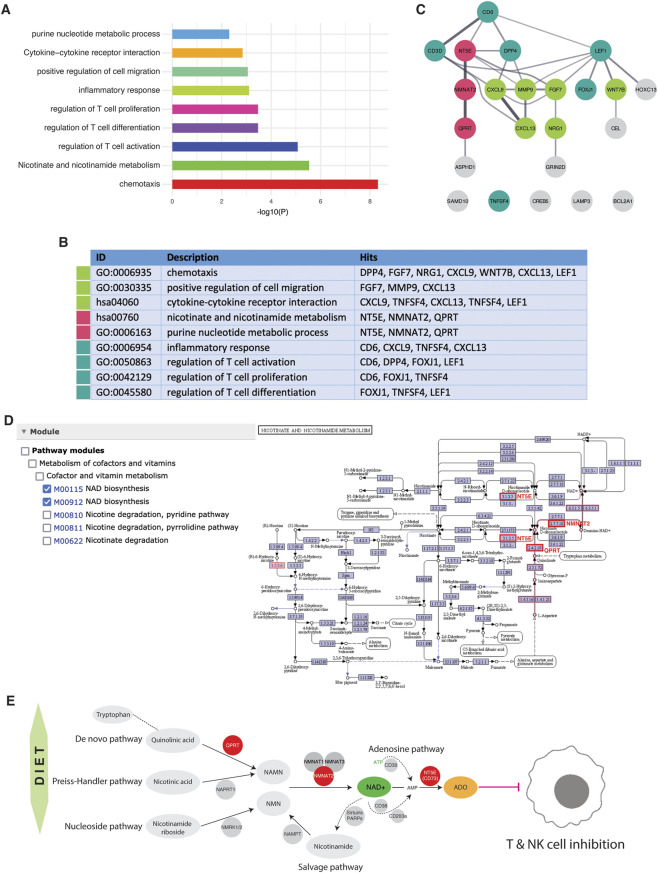
Functional analysis of TME-Metab prognostic index. **(A)** Bar plot and **(B)** Table showing the enrichment of Gene Ontology (GO) and KEGG pathways using ShinyGO 0.80. **(C)** Protein-protein interaction (PPI) network illustrates protein-level interactions of TME-Metab prognostic index (TMPI) (interaction score = 0.4). **(D)** Illustration of KEGG pathway “Nicotinate and Nicotinamide Metabolism” using the KEGG PATHWAY database. **(E)** Illustration of Nicotinamide adenine dinucleotide (NAD) metabolism and adenosine pathway from medical literature. Red indicates the enzymes that are part of the TMPI genes.

To explore the biological processes underlying TMPI-defined phenotypes, functional enrichment analysis of the 23-gene signature was performed. The analysis revealed three major functional themes: cell migration and chemotaxis, regulation of T-cell activation and differentiation, and nicotinate and nicotinamide metabolism (Figures 7A,B). Protein-protein interaction analysis demonstrated close connectivity among these pathways, suggesting coordinated interactions between metabolic processes and immune regulation (Figure 7C). Notably, three key genes—NMNAT2, QPRT, and NT5E—were consistently associated with the nicotinate and nicotinamide metabolism pathway, highlighting a potential role for NAD-related processes within TMPI-defined phenotypes (Figure 7D).

NAD biosynthesis occurs through three principal pathways: the *de novo*, Preiss-Handler, and nucleoside (salvage) pathways (Figure 7E) (Navas and Carnero, 2021; Mogol et al., 2023; Yaku et al., 2018; Navas and Carnero, 2022; Fang et al., 2017; Burgos, 2011; Allard et al., 2020). Within this framework, QPRT primarily functions in the *de novo* pathway, whereas NMNAT2 catalyzes a critical step shared across multiple NAD biosynthetic routes, including salvage and recycling processes. In addition to NAD synthesis, its degradation contributes to downstream signaling. NAD^+^ can be metabolized by enzymes such as PARPs, sirtuins, and the ectoenzyme CD38, generating ADP-ribose (ADPR). ADPR is subsequently converted to AMP via CD203a in the non-canonical pathway, while in the canonical pathway, ATP is sequentially hydrolyzed to AMP by CD39. In both pathways, AMP is ultimately converted to extracellular adenosine by CD73 (NT5E). Extracellular adenosine is a well-established immunomodulatory molecule and has been implicated in the suppression of T- and NK-cell activity within the tumor microenvironment. These pathways highlight a potential link between NAD metabolism and immunosuppressive signaling in TMPI-defined phenotypes.

### Cellular sources and immune associations of NAD metabolism genes in breast cancer

**FIGURE 8:**
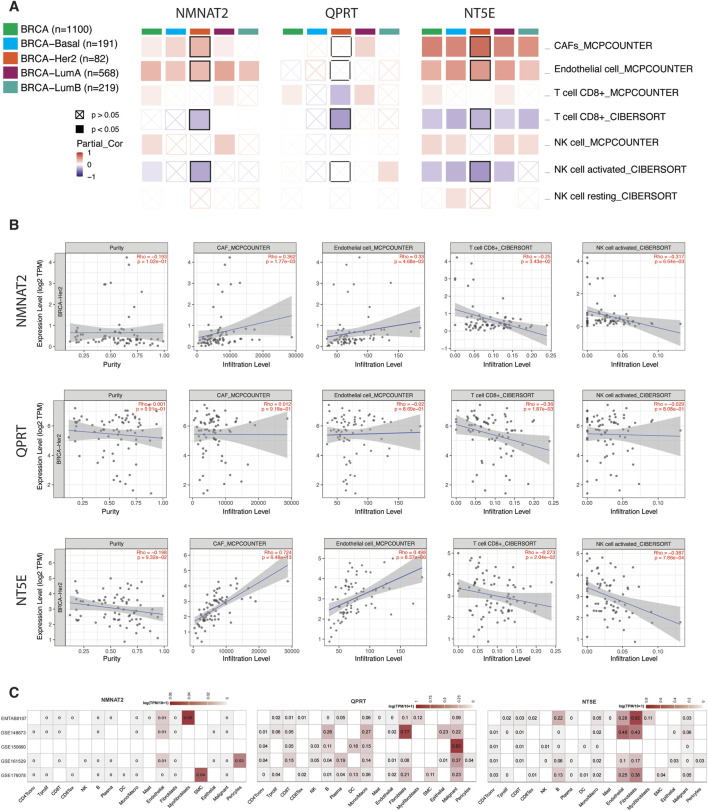
Expression dynamics of NMNAT2, QPRT and NT5E in breast cancer. **(A)** Spearman’s correlation between NMNAT2, QPRT and NT5E and infiltration of various immune cells in the TCGA BRCA using TIMER database. **(B)** Correlation graph showing the significant association between expression of NMNAT2, QPRT, and NT5E and tumor purity/various immune cells in TCGA BRCA HER-2+ subtype. **(C)** Expression analysis of NMNAT2, QPRT and NT5E in various cell types of five breast cancer single-cell datasets using the TISCH database.

To further define the cellular context of NAD metabolism within the tumor microenvironment, we examined the association of key genes (NMNAT2, QPRT, and NT5E) with tumor purity and immune/stromal cell infiltration using TIMER analysis in the TCGA-BRCA cohort (Figures 8A,B). NMNAT2 and NT5E showed strong positive correlations with fibroblasts and endothelial cells, alongside negative associations with T cells and activated NK cells, particularly in HER2-positive subtypes. QPRT exhibited a weaker but consistent pattern, with a negative association with CD8^+^ T cells and a modest correlation with tumor purity. These observations were further supported by single-cell analyses across multiple breast cancer datasets (Figure 8C). NMNAT2 expression was enriched in endothelial cells, pericytes, myofibroblasts, and stem-like cells, while NT5E was predominantly expressed in endothelial cells and fibroblasts. In contrast, QPRT showed enrichment across malignant cells, fibroblasts, and monocyte/macrophage populations. Together, these findings indicate that NAD metabolism-associated genes are primarily expressed in tumor and stromal compartments and are consistently associated with reduced infiltration of T and NK cells. This cellular distribution supports a link between NAD metabolic activity and the establishment of an immunosuppressive tumor microenvironment in TMPI-defined high-risk phenotypes.

### Validation of NMNAT2, QPRT, and NT5E expression in breast cancer

**FIGURE 9:**
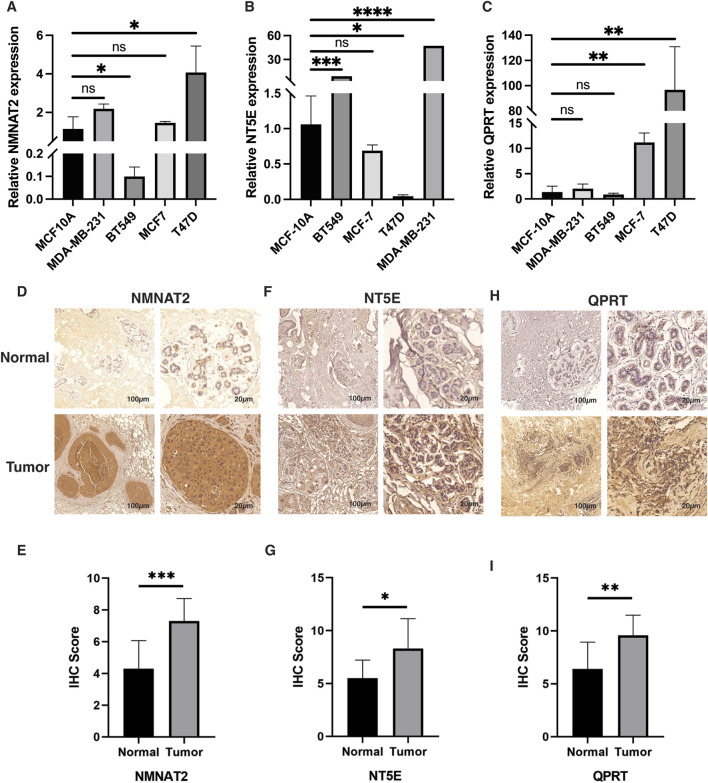
Validation of NMNAT2, QPRT and NT5E in breast cancer. **(A–C)** Bar plots show the mRNA expression levels of NMNAT2, QPRT, and NT5E in normal breast cells (MCF-10A) and breast cancer cells (MCF-7, BT549, MDA-MB-231, and T47D). The data represent the mean ± SEM (standard error of the mean) of n = 3 independent experiments (independent biological replicates) for each condition. Two-tailed unpaired T-test; *P < 0.05; **P < 0.01; ***P < 0.001; ****P < 0.0001; ns, not significant. Immunohistochemistry (IHC) staining of **(D)** NMNAT2, **(F)** QPRT, and **(H)** NT5E in normal and cancerous breast cancer tissues (n = 10). Bar plots comparing the expression levels (IHC quantification) of **(E)** NMNAT2, **(G)** QPRT, and **(I)** NT5E in cancerous and para-cancerous normal breast cancer tissues (n = 10). P values are shown as: *P < 0.05; **P < 0.01; ***P < 0.001.

We assessed the mRNA expression levels of NMNAT2, QPRT, and NT5E using qPCR in breast cancer cell lines (MCF-7, MDA-MB-231, T47D, and BT549) and normal breast epithelial cells (MCF-10A) (Figures 9A–C). NMNAT2 and QPRT were significantly upregulated in estrogen receptor-positive (ER+) breast cancer cells (MCF-7 and T47D), whereas NT5E expression was markedly elevated in triple-negative breast cancer (TNBC) cell lines (MDA-MB-231 and BT549). At the protein level, immunohistochemical (IHC) analysis demonstrated significantly higher expression of NMNAT2, QPRT, and NT5E in breast cancer tissues compared to adjacent normal tissues (n = 10) (Figures 9D–I). These findings confirm the differential expression of key TME–metabolism genes across breast cancer subtypes and support their potential role in tumor progression.

### TMPI and NAD metabolism genes associate with immune microenvironment states and differential immunotherapy response

**FIGURE 10:**
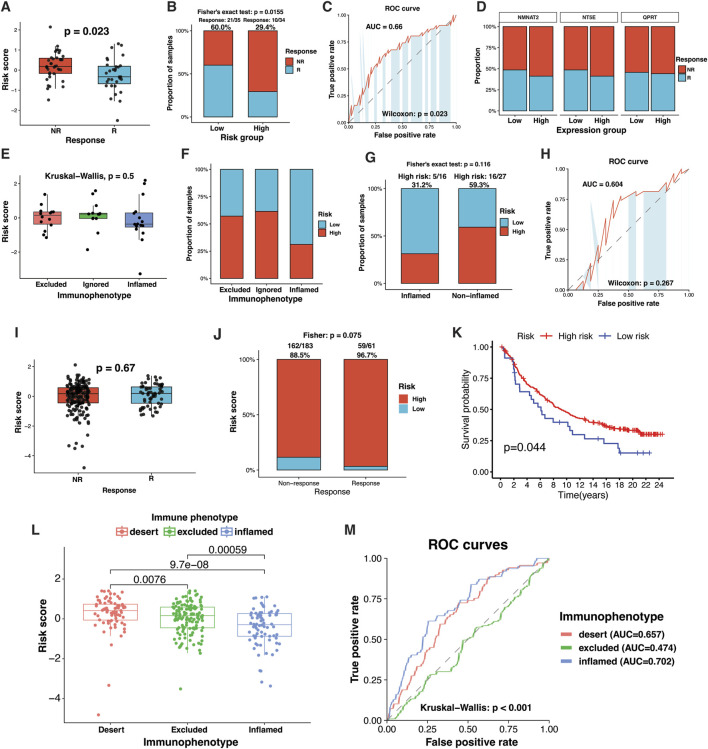
TMPI associations with immunotherapy response and tumor immune microenvironment phenotypes. **(A)** Box plot showing comparison of TMPI risk scores between responders and non-responders in the anti -PD-1-treated breast cancer immunotherapy cohort. Statistical significance was evaluated using the Wilcoxon rank-sum test. **(B)** Proportion of responder and non-responder patients within TMPI high- and low-risk subgroups in the breast cancer cohort. Statistical significance was assessed using the chi-square or Fisher’s exact test, as appropriate. **(C)** ROC curve evaluating the predictive performance of TMPI for immunotherapy response in the breast cancer cohort. **(D)** Proportion of responder and non-responder patients within high- and low-expression subgroups of NMNAT2, NT5E, and QPRT in the breast cancer cohort. **(E)** Box plot comparing TMPI risk scores across tumor immune microenvironment (TME) phenotypes (inflamed, excluded, and desert) in the breast cancer cohort. Statistical significance was evaluated using the Kruskal–Wallis test with pairwise Wilcoxon comparisons. **(F)** Distribution of TMPI high- and low-risk groups across inflamed and non-inflamed TME phenotypes in the breast cancer cohort. **(G)** Proportion of inflamed and non-inflamed tumors within TMPI risk subgroups in the breast cancer cohort. Statistical significance was assessed using the chi-square or Fisher’s exact test, as appropriate. **(H)** ROC curve evaluating the predictive performance of TMPI for inflamed versus non-inflamed TME classification in the breast cancer cohort. **(I)** Box plot comparing TMPI risk scores between responders and non-responders in the IMvigor210 anti -PD-L1-treated cohort. Statistical significance was evaluated using the Wilcoxon rank-sum test. **(J)** Distribution of immunotherapy response categories across TMPI risk subgroups in the IMvigor210 cohort. Statistical significance was assessed using the chi-square test. **(K)** Kaplan–Meier survival analysis comparing overall survival between TMPI high- and low-risk groups in the IMvigor210 cohort using the log-rank test. **(L)** Box plot showing the association between TMPI risk scores and tumor immune phenotypes (inflamed, excluded, and desert) in the IMvigor210 cohort. Statistical significance was evaluated using the Kruskal–Wallis test with pairwise Wilcoxon comparisons. **(M)** ROC curves evaluating the predictive performance of TMPI for inflamed and desert TME phenotype classification in the IMvigor210 cohort. Abbreviations: CR, complete response; PR, partial response; SD, stable disease; PD, progressive disease; ROC, receiver operating characteristic; TME, tumor microenvironment.

The association of TMPI with immunotherapy response and tumor immune microenvironment (TIME) characteristics was evaluated across immunotherapy cohorts (Figure 10). In the breast cancer cohort treated with anti-PD-1 therapy (Pembrolizumab), non-responders exhibited higher TMPI scores compared to responders, with an increased proportion of high-risk patients observed in the non-responder group (Figures 10A,B). ROC analysis demonstrated moderate predictive performance (AUC ≈0.66; Figure 10C). Non-response patients showed only a modest increased in NMNAT2 and NT5E expression (Figure 10D).

TMPI scores showed variable associations with immune phenotypes in the breast cancer cohort (Figures 10E–H). Although no significant differences were observed across inflamed, excluded, and ignored (desert) groups based on direct comparison of TMPI scores (Figure 10E), distribution analyses suggested a trend toward lower TMPI values in inflamed tumors. Proportion analysis indicated that a smaller fraction of inflamed tumors was classified as high risk compared to non-inflamed tumors (Figures 10F,G). Consistently, when inflamed and non-inflamed groups were compared, 31.2% of inflamed tumors versus 59.3% of non-inflamed tumors were classified as high risk, although this difference did not reach statistical significance (Figure 10G). ROC analysis for predicting inflamed versus non-inflamed status yielded modest performance (AUC ≈0.60; Figure 10H). Together, these findings provide moderate evidence, probably due to low sample size, for an inverse association between TMPI and inflamed tumor microenvironment states in the breast cancer cohort.

To further validate these findings in an independent larger immunotherapy cohort and across a different cancer type, we analyzed the IMvigor210 dataset treated with anti-PD-L1 therapy (Figures 10I–M). In the IMvigor210 cohort, TMPI showed distinct associations with response, survival, and tumor immune phenotypes. Comparison of TMPI scores between responders and non-responders did not reveal a significant difference based on boxplot analysis (Figure 10I). However, proportion analysis indicated a higher frequency of high-risk tumors among responders compared to non-responders (96.7% vs. 88.5%), demonstrating a trend opposite to that observed in the anti–PD-1 cohort (p = 0.075; Figure 10J). Survival analysis using the optimal cutoff demonstrated a significant association between TMPI and overall survival (p = 0.044; Figure 10K). Notably, while most patients were classified as high risk, the smaller low-risk subgroup exhibited poorer survival outcomes. Analysis of tumor immune phenotypes showed a significant association between TMPI and TME states (Figure 10L). TMPI scores were highest in immune-desert tumors, followed by immune-excluded tumors, and lowest in inflamed tumors (Figure 10L). ROC analysis demonstrated improved discriminative performance compared to the breast cancer cohort, with AUC values of approximately 0.657 for desert and 0.702 for inflamed phenotype classification (Figure 10M).

**FIGURE 11:**
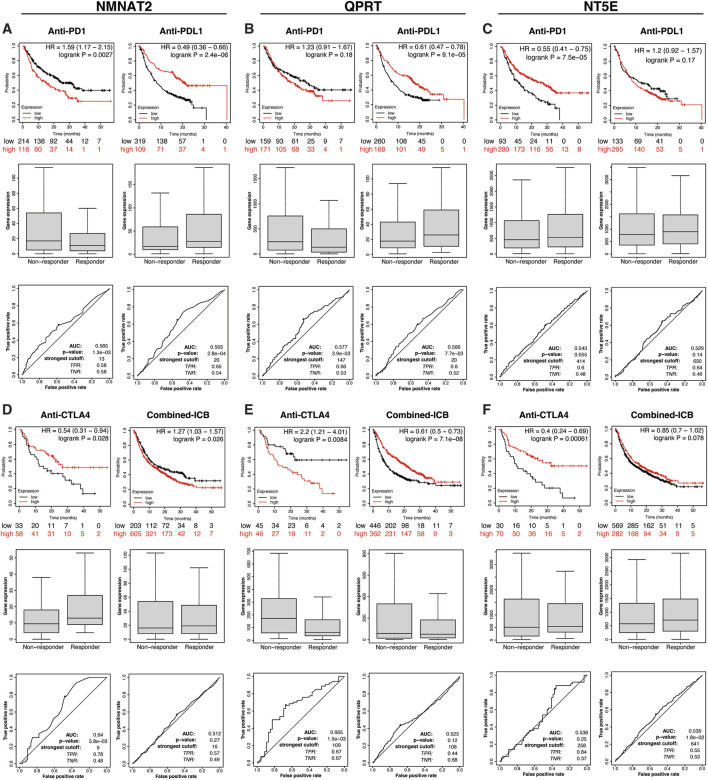
Individual impact of expression of NMNAT2, QPRT, and NT5E on response to immunotherapy Evaluation of response to anti-PD-1, anti-PD-L1, anti-CTLA, and combined immune checkpoint blockade between cancer patients stratified by the expression of NMNAT2 **(A,D)**, QPRT **(B,E)**, and NT5E **(C,F)** was demonstrated by Kaplan-Meier curves of overall survival, bar plots of response rate, and receiver operating characteristic (ROC) graphs showing area under curve (AUC) values for response prediction.

Given the strong association of NAD metabolism-related genes (NMNAT2, QPRT, and NT5E) with reduced CD8^+^ T-cell and NK-cell activity, we further explored their relationship with immunotherapy outcomes using datasets from the ROC Plotter platform, encompassing 1,434 cancer patients. Elevated expression of NMNAT2 and QPRT was associated with reduced responsiveness to anti-PD-1 therapy but showed improved response trends in anti–PD-L1-treated cohorts, as supported by survival analyses and response distribution comparisons (Figures 11A,B). ROC analyses further indicated statistically significant differences in gene expression between responders and non-responders; however, the corresponding AUC values were modest, suggesting limited response predictive performance (Figures 11A,B). In contrast, NT5E exhibited an inverse association with response to both anti-PD-1 and anti-PD-L1 therapies (Figure 11C). The effects of these genes on anti-CTLA-4 therapy and combined-ICB response varied across datasets (Figures 11D–F). Overall, TMPI was consistently associated with immune-excluded and immune-desert TME states across cohorts and showed therapy-dependent associations with immunotherapy response, while NAD metabolism-related genes demonstrated concordant patterns in independent datasets.

## Discussion

Breast cancer is a heterogeneous disease characterized by significant intertumoral and intratumoral variability, which poses diagnostic and therapeutic challenges (Turashvili and Brogi, 2017; Polyak, 2011). Clinical staging, pathological grading, and molecular subtyping have been widely adopted to address this complexity and guide clinical treatment (Guo et al., 2023). Molecular subtyping of breast cancer has enabled the identification and application of novel therapeutics, including hormone-based and anti-HER2 targeted therapies (Yersal and Barutca, 2014; Jallah et al., 2023). However, these classification systems have limitations, as they do not fully account for heterogeneity arising from other cancer hallmarks, particularly TME factors and associated metabolic processes (Atallah et al., 2023). In this study, we adopted a novel strategy to identify metabolic factors that are either upregulated by cancer cells or integrated within the TME, thereby influencing breast cancer prognosis.

**FIGURE 12:**
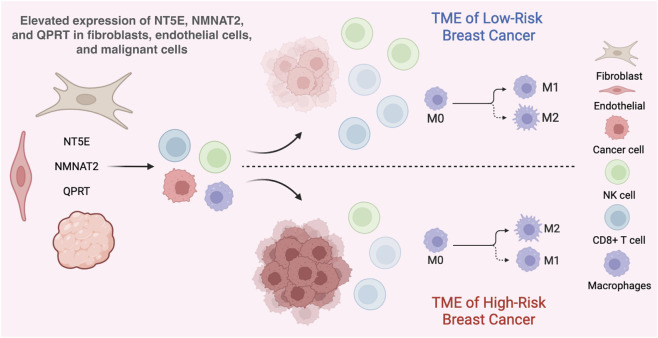
Overview of the classification of breast cancer patients based on the metabolically active factors of the tumor microenvironment. A comprehensive analysis of the transcriptomic data of breast cancer revealed two BRCA subgroups that varied in their TME composition and prognosis. Upregulation of NMNAT2, QPRT, and NT5E in stromal (predominantly endothelial and fibroblasts) and malignant cells suppresses the infiltration and activity of CD8 + T and NK cells within the breast cancer TME. Furthermore, the TME lacking CD8 + T and NK cells exhibited heightened M2 polarization, correlating with an unfavorable prognosis.

A high-risk phenotype of breast cancer was identified based on a core group of 36 interconnected crosstalk genes, primarily composed of TME-related genes together with a subset of metabolic genes. These genes demonstrated enhanced nicotinate metabolism and were associated with reduced CD8^+^ T-cell and NK-cell infiltration within the TME (Figure 12). Previous transcriptomic studies have similarly identified immune-based breast cancer subtypes characterized by varying levels of CD8^+^ T-cell infiltration and PD-L1-associated immune activity, with immune-high phenotypes being enriched in TNBC and HER2 subtypes (Yao et al., 2021; Safonov et al., 2017). Consistent with these observations, clustering based on the 36 crosstalk genes identified a Cluster one subgroup enriched in TNBC and HER2+ tumors that exhibited increased CD8^+^ T-cell infiltration and improved prognosis.

Although the original crosstalk network was observed across breast cancer subtypes, its prognostic impact was most evident in HER2+ and Luminal B tumors. Stratified survival analyses demonstrated significant survival separation between TME–metabolism subtypes in HER2+ and Luminal B breast cancers, whereas basal-like and Luminal A tumors showed limited prognostic stratification. Further refinement of the interaction network generated the 23-gene TMPI signature, which retained the original immune–metabolic interaction profile but shifted the high-risk phenotype toward HER2-enriched and Luminal B contexts. Given that many Luminal B tumors co-express HER2, these findings suggest that TMPI-defined subtypes may preferentially capture metabolically rewired and immune-suppressed HER2-associated tumor states rather than generalized immune activation alone (Höller et al., 2023).

Consistent with our findings, recent studies have shown that metabolically active breast cancer states enriched in Luminal B and HER2 subtypes are characterized by proliferative and stromal-associated tumor microenvironments involving coordinated signaling between tumor, stromal, and myeloid cells, whereas immune-activated states enriched in Luminal A tumors demonstrate enhanced T/NK-cell interactions and antigen-presentation signaling (Cheng et al., 2025). Together, these findings support the notion that distinct TME architectures underlie subtype-specific metabolic programs and may influence the prognostic impact of TMPI. Notably, the high-risk TMPI subtype was not completely devoid of CD8^+^ T cells but instead exhibited features suggestive of impaired or spatially restricted T-cell activity within the TME. Overall, TMPI-defined subtypes may provide clinically relevant biomarkers for prognostic stratification and characterization of immune-metabolic heterogeneity in biologically aggressive breast cancer contexts.

The infiltration and functional dynamics of CD8^+^ T cells within the TME are critical determinants of prognosis and immunotherapy response. Immune-inflamed tumors generally exhibit improved therapeutic responsiveness, whereas immune-excluded and immune-desert phenotypes are associated with impaired cytotoxic lymphocyte activity and reduced anti-tumor immunity (Galon and Bruni, 2019; Gruosso et al., 2019; Li et al., 2021; Loi et al., 2014; Yao et al., 2021; Safonov et al., 2017). Consistent with these observations, the TMPI low-risk subtype demonstrated increased CD8^+^ T-cell infiltration and favorable prognosis.

Nicotinamide adenine dinucleotide (NAD) metabolism is increasingly recognized as an important factor in cancer biology, including breast cancer (Navas and Carnero, 2021; Mogol et al., 2023). NAD metabolism plays a central role in maintaining cellular NAD pools for both redox and non-redox reactions. Redox reactions, including glycolysis, the tricarboxylic acid (TCA) cycle, oxidative phosphorylation, and serine biosynthesis, are critical for tumor progression (Navas and Carnero, 2021; Yaku et al., 2018). Non-redox reactions involve inflammatory signaling, DNA repair, post-translational modifications, cell signaling, senescence, and apoptosis (Navas and Carnero, 2022; Fang et al., 2017). In these processes, NAD^+^ serves as a substrate for enzyme families such as sirtuins, poly (ADP-ribose) polymerases (PARPs), and cyclic ADP-ribose polymerases (cADPRs) (Navas and Carnero, 2021; Fang et al., 2017). NAD degradation by these enzymes produces nicotinamide (NAM), which is subsequently converted to nicotinamide mononucleotide (NMN) by nicotinamide phosphoribosyltransferase (NAMPT) (Burgos, 2011).

The oncogenic role of NAMPT in breast cancer has been reported previously, with elevated expression associated with larger tumor size, lymph node metastasis, advanced tumor stages, and hormone receptor expression (Zhou et al., 2018). Additionally, treatment with fluvastatin has been shown to upregulate NAMPT, and combined inhibition of NAMPT (KPT-9274) and fluvastatin demonstrated synergistic effects in reducing metastatic tumor burden (Mogol et al., 2023). The absence of NAMPT in our TMPI signature suggests a comparable contribution of the salvage pathway across TMPI subtypes. In contrast, our findings highlight the upregulation of QPRT and NMNAT2, indicating a potential predominance of the *de novo* NAD biosynthesis pathway. Previous analyses of TCGA BRCA RNA-seq data have also demonstrated the prognostic relevance of these genes (Zhou et al., 2018; Zhou et al., 2022). NMNAT2 has been linked to colorectal cancer progression, although its role in breast cancer remains less defined (Qi et al., 2018). NMNAT enzymes, particularly NMNAT2, play a critical role in regulating NAD synthesis, as multiple NAD biosynthesis pathways converge at this step (Buonvicino et al., 2018). Experimental studies have shown that inhibition of NAMPT and NMNAT2 can lead to NAD depletion and impaired tumor cell viability, highlighting its potential therapeutic relevance. QPRT has also been associated with poor prognosis in breast cancer (Yu et al., 2021; Zhou et al., 2022; Yan et al., 2023). Our findings further support the dynamic expression and potential clinical significance of these genes, although additional studies are needed to clarify their mechanistic roles.

Our analysis also highlighted the importance of NT5E (CD73), which contributes to extracellular adenosine production through NAD degradation pathways (Allard et al., 2020). Dysregulated CD73 expression, often referred to as a metabolic immune checkpoint, has been reported in multiple cancers, including breast cancer (Neo et al., 2019). CD73 is associated with tumor progression, immune evasion, and therapy resistance through its role in adenosine-mediated signaling. Previous studies have demonstrated its involvement in immune and stromal compartments, including regulatory T cells, NK cells, cancer-associated fibroblasts, and endothelial cells, where it contributes to suppression of T-cell responses (Schuler et al., 2014; Turcotte et al., 2015; Yu et al., 2020; Costa et al., 2018). CD73-mediated adenosine signaling has also been implicated in modulating lymphocyte trafficking and tumor angiogenesis (Thompson et al., 2004; Takedachi et al., 2008; Wang et al., 2011; Wang et al., 2013; Allard et al., 2014). These observations are consistent with our findings, in which NT5E expression was associated with immunosuppressive features of the TME.

The role of NAD metabolism-related genes in shaping immunotherapy response is increasingly recognized. In this study, TMPI was consistently associated with immune-excluded and immune-desert TME states across immunotherapy cohorts, while its associated genes (NMNAT2, QPRT, and NT5E) were linked to reduced CD8^+^ T-cell and NK-cell activity in the TCGA-BRCA cohort. These findings are biologically plausible, as NAD metabolism and adenosine signaling pathways are known to regulate T-cell activation, metabolic fitness, exhaustion, and immune evasion within the TME (Navas and Carnero, 2021; Fang et al., 2017). In particular, NAD-dependent pathways involving NAMPT, PARPs, sirtuins, and CD73-mediated adenosine production have been implicated in suppression of anti-tumor immunity and establishment of immunosuppressive microenvironments (Navas and Carnero, 2021; Fang et al., 2017; Neo et al., 2019; Schuler et al., 2014; Turcotte et al., 2015; Yu et al., 2020; Costa et al., 2018; Thompson et al., 2004; Takedachi et al., 2008; Wang et al., 2011; Wang et al., 2013; Allard et al., 2014). Previous studies have also demonstrated that immune-excluded and immune-desert tumors are frequently characterized by stromal remodeling, impaired cytotoxic lymphocyte infiltration, and metabolically restrictive microenvironments that limit effective T-cell function (Galon and Bruni, 2019; Gruosso et al., 2019; Li et al., 2021). In this context, the association of TMPI with non-inflamed immune phenotypes suggests that the signature may reflect broader immunometabolic programs linked to dysfunctional or spatially restricted anti-tumor immune activity rather than immune cell abundance alone.

Interestingly, distinct patterns were observed between anti-PD-1 and anti-PD-L1-treated cohorts. In the breast cancer anti–PD-1 cohort, elevated TMPI scores were associated with poorer response and enrichment of non-inflamed TME states. In contrast, the IMvigor210 anti-PD-L1 cohort demonstrated a different response distribution pattern despite maintaining strong associations with immune-desert and immune-excluded phenotypes. Similar therapy-dependent trends were also observed for NMNAT2 and QPRT in independent ROC Plotter pan-cancer datasets. One possible explanation is that PD-1 blockade primarily acts through reinvigoration of exhausted PD-1^+^ T cells, whereas the high-risk TMPI phenotype was characterized by reduced CD8^+^ T-cell and NK-cell activity together with non-inflamed TME states (Ai et al., 2020; Tumeh et al., 2014). In contrast, PD-L1 is broadly expressed on tumor cells, stromal cells, dendritic cells, macrophages, and other myeloid populations within the tumor microenvironment, and PD-L1 blockade may therefore influence a wider range of suppressive cellular interactions beyond T-cell intrinsic signaling alone (Ai et al., 2020; Tumeh et al., 2014; Lin et al., 2018). Previous studies have also demonstrated that immune-excluded and immune-desert tumors are characterized by stromal remodeling, impaired cytotoxic lymphocyte infiltration, and myeloid-mediated immunosuppressive programs that can influence responsiveness to immune checkpoint blockade (Galon and Bruni, 2019; Gruosso et al., 2019; Li et al., 2021; Hegde and Chen, 2020). Collectively, these findings suggest that NAD metabolism-associated immune states may interact differently with PD-1 and PD-L1 blockade contexts, potentially reflecting differences in checkpoint biology and microenvironmental regulation.

Overall, TMPI demonstrated more consistent associations with tumor immune microenvironment phenotypes than with direct immunotherapy response prediction, particularly through enrichment of immune-desert and immune-excluded states across independent cohorts. In contrast, although NMNAT2, QPRT, and NT5E showed significant associations with immunotherapy outcomes in independent pan-cancer datasets, their modest AUC values suggest limited utility as standalone predictive biomarkers. Collectively, these findings support the notion that TMPI and NAD metabolism-associated genes may primarily reflect broader immunometabolic states that shape anti-tumor immune activity and influence immunotherapy responsiveness in a context-dependent manner.

## Conclusion

In summary, this study highlights the interplay between tumor metabolism and the tumor microenvironment in shaping breast cancer progression. By integrating metabolic and TME-related gene signatures, we identified TMPI as a prognostic model that captures clinically relevant dimension of immune-metabolic crosstalk. TMPI-defined high-risk phenotypes were characterized by enhanced metabolic activity, stromal enrichment, reduced T- and NK-cell-associated immune activity, and enrichment of non-inflamed tumor microenvironment states. The involvement of NAD metabolism-related genes, including NMNAT2 and QPRT, together with the adenosine-generating enzyme NT5E, suggests a potential metabolic axis contributing to immune modulation and tumor progression. These findings provide a framework for understanding how metabolic reprogramming intersects with immune regulation in breast cancer and may support improved prognostic stratification and therapeutic targeting, particularly in HER2+ and Luminal B subtypes.

## Data Availability

The datasets supporting the conclusions of this article are available in The Cancer Genome Atlas (https://portal.gdc.cancer.gov/) and GEO (https://www.ncbi.nlm.nih.gov/gds) repositories. Further information and requests for resources should be directed to and will be fulfilled by Lead Contact, Jiacai Ye, 21376689@qq.com.
